# Interaction Between PEDV and Its Hosts: A Closer Look at the ORF3 Accessory Protein

**DOI:** 10.3389/fvets.2021.744276

**Published:** 2021-09-10

**Authors:** Yuparat Jantraphakorn, Ratchanont Viriyakitkosol, Anan Jongkaewwattana, Challika Kaewborisuth

**Affiliations:** ^1^Virology and Cell Technology Research Team, National Center for Genetic Engineering and Biotechnology (BIOTEC), National Science and Technology Development Agency, Pathumthani, Thailand; ^2^Faculty of Veterinary Science, Chulalongkorn University, Bangkok, Thailand

**Keywords:** PEDV, ORF3, virus replication, pathogenesis, virus and host interaction, vaccine development

## Abstract

Porcine epidemic diarrhea virus (PEDV) is a causative agent of a highly contagious enteric disease in swine of all ages, leading to severe economic losses for the swine industry in many countries. One of the most effective approaches in controlling PEDV infection is vaccination. The ORF3 accessory protein has been proposed as a crucial viral virulence factor in a natural host. However, due to the lack of an extensive comparative study of ORF3, exactly how the ORF3 takes part in virus replication and pathogenesis as well as its role in host-virus interaction is unclear. In this review, we aim to discuss the current knowledge of ORF3 concerning its dispensability for viral replication *in vitro*, ability to modulate host responses, contribution to virus pathogenicity, and research gaps among ORF3 functional studies. These will be beneficial for further studies to a better understanding of PEDV biology and PEDV vaccine development.

## Introduction

Porcine epidemic diarrhea virus (PEDV) is an enteric pathogen that has spread in the swine population. PEDV infection causes severe watery diarrhea, dehydration, vomiting, and death, particularly in neonatal piglets, resulting in massive economic losses in pig industries worldwide, particularly in the United States, China, South Korea, and Thailand (1–4). According to the phylogenetic analysis, PEDV has been classified into two major groups, namely genotype 1 (G1) and genotype 2 (G2) (5). The G1 group could be divided into two sub-genotypes: G1a and G1b. The classical PEDV strains CV777 and DR13 are the G1 representatives (6). During the 1980-the 2000s, the G1a PEDV had caused outbreaks in Asia (7). Despite its moderate virulence, vaccines against this diarrheal disease had been developed and used in many countries, including Japan, China, and South Korea (8). The highly virulent G2 strain has emerged in China in 2010 and spread to many countries worldwide (2, 3), with the mortality rate in nursing piglets almost 100% (9). Although various G1a-based vaccines have been used to control the outbreaks, their efficacy against these highly virulent strains is minimal (10). Vaccines specifically designed for the G2 genotype are thus necessary to effectively control the ongoing PEDV epidemics.

**Figure 1:**
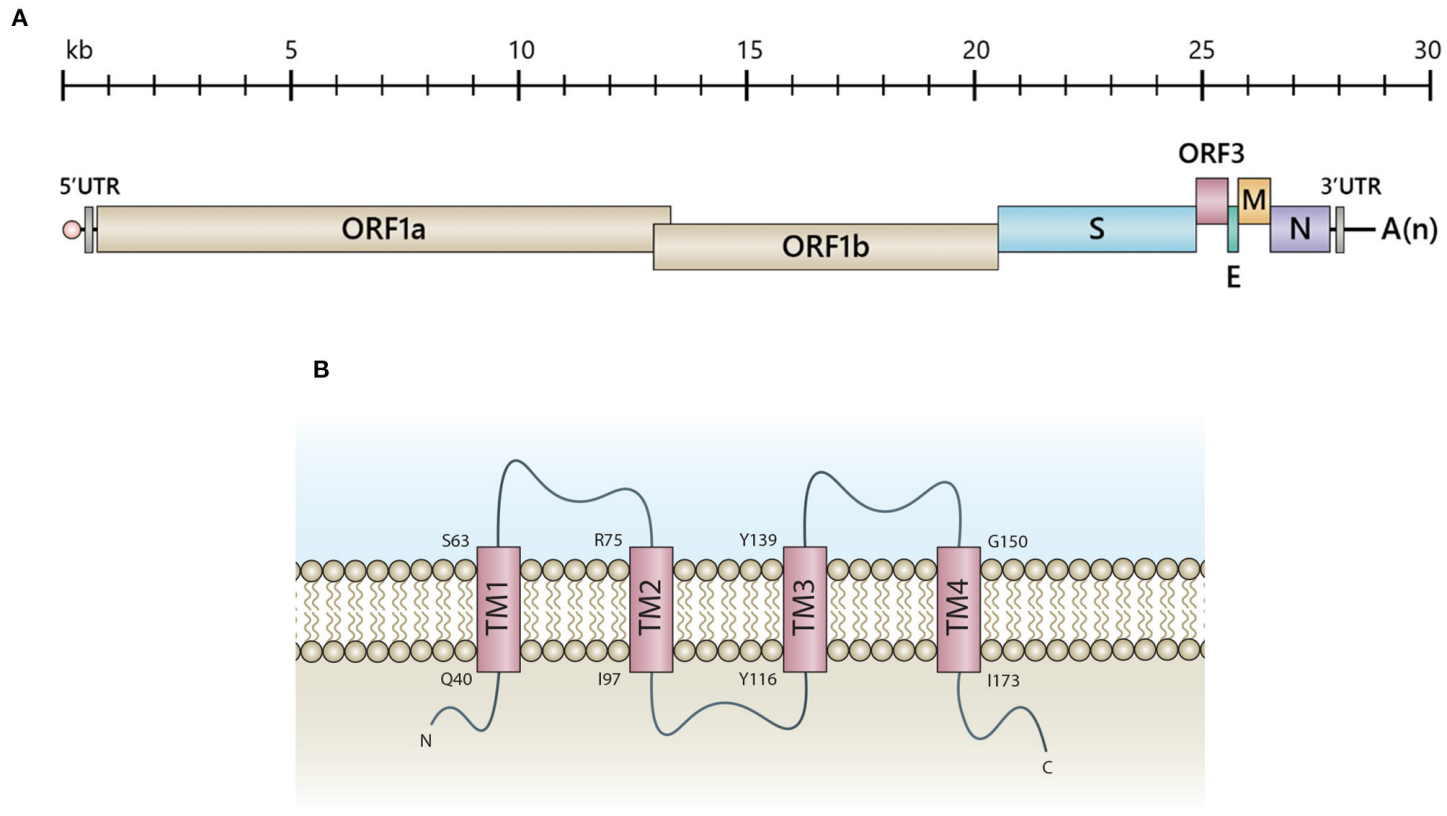
Schematic representation of PEDV genomic RNA and the predicted ORF3 structure. **(A)** The PEDV genome consists of 5'-capped UTR (5'UTR), ORF1a, and ORF1b followed by the genes encoding the spike (S), accessory protein (ORF3), envelope (E), membrane protein (M) and nucleoprotein (N), and 3'polyadenylated UTR (3'UTR). **(B)** Illustration of a predicted ORF3 structure proposed by computational modeling and transmembrane prediction programs (11). Transmembrane (TM) domains include TM1 (aa positions 40-63), TM2 (aa positions 75-97), TM3 (aa positions 116-139) and TM4 (aa positions 150-173). Q, Glutamine; S, Serine; R, Arginine; I, Isoleucine; Y, Tyrosine; G, Glycine.

Belonging to the genus *Alphacoronavirus*, PEDV is an enveloped virus bearing positive-sense single-stranded RNA of approximately 28 kb in length. The viral genome comprises at least seven open reading frames (Figure 1A) encoding two polyproteins, pp1a and pp1ab, which can be processed into 16 non-structural proteins (nsps), four structural proteins (spike, S; envelope, E; membrane, M and nucleocapsid, N), and only one accessory protein, the ORF3 (12). For decades, numerous studies have been carried out to gain more insights into various aspects of PEDV, including basic virology, pathogenesis, immune responses and vaccine design. Despite the rapidly accumulating data of the coronavirus structural proteins, those related to the accessory proteins are relatively limited. It has been demonstrated that PEDV ORF3 participates in increased virus infection and lesion in the swine intestinal tract (11, 13). The defective PEDV ORF3 with deletion at a C-terminus acquired after virus adaptation in cell culture appeared to reduce virus virulence (13, 14). However, the evidence so far could not delineate how the ORF3 governs virus pathogenesis and virus replication *in vitro* and *in vivo*. Many questions remain unanswered and need comprehensive studies to understand the functions of this protein.

## The Role of PEDV ORF3 in Virus Virulence

The PEDV ORF3 [675 nucleotides; 224 amino acids (aa)] is a viroporin, consisting of four transmembrane domains possessing a potassium ion channel activity (11) (Figure 1B). Viral proteins bearing ion channel activity, including E proteins of MHV (15), SARS-CoV (16), and IBV (17), have been reported to play a role in pathogenesis, virus assembly, and release. Studies performed in *Xenopus laevis* oocytes and yeast cells expressing transmembrane-truncated PEDV ORF3 demonstrated that the constructs bearing deleted amino acid at positions 82-98 and 151-172 completely lost the channel activity (11), which might be associated with the reduced pathogenicity of PEDV strain DR13 (aa82-99 deletion) in piglets (18). However, the evidence showing that the ion channel activity of ORF3 is necessary for PEDV replication is still lacking. Most of the available data are based on studies in ORF3a of SARS-CoV. The ion channel activity of SARS-CoV ORF3a has been shown to play a role in vial pro-apoptotic property (19), virus replication, and pathogenesis in an animal model (20). Nevertheless, the SARS-CoV ORF3a's ion activity is not related to the induction of IL-1β, virus replication and virus virulence in cell culture (20, 21). The study also demonstrated a functional compensation between ORF3a and other viroporins on virus viability and disease severity, impacting the rational vaccine design (20). However, it should be noted that PEDV ORF3 and SARS-CoV ORF3a are quite remarkably distinct in terms of protein structure and function. PEDV ORF3 likely may not utilize its ion channel activity in the same manner as observed in SARS-CoV or other coronaviruses.

Wild-type PEDV and cell culture adapted PEDV (ca-PEDV) have been shown to exert different degrees of disease severity in the infected hosts. Bernasconi et al. (22) showed that only 5 out of 21 newborn piglets inoculated with ca-PEDV had mild watery diarrhea, and no other clinical signs were observed. Likewise, piglets orally inoculated with KPEDV-9, a highly adapted variant, did not have signs of diarrhea or other clinical symptoms (23). PEDV DR13 strain that was serially passaged in Vero cells also lost virus pathogenicity (24). When the genetic constellation of wild-type and attenuated PEDVs were analyzed and compared, it is evident that PEDV ORF3 gene derived from attenuated PEDVs, but not the virulent variant, displays shortened or truncated ORF3 with 51 nucleotide deletions (18), suggesting that the PEDV ORF3 might involve in disease severity. A recent study by Wang et al. (13) demonstrated that ORF3 might play a crucial role in viral virulence. By swapping the S gene derived from the virulent strain into the genome of the avirulent strain, the authors showed that the chimeric virus did not alter the infectivity and invasiveness of attenuated strain, suggesting that S alone is not the only satisfactory factor obligated for virus virulence. The authors later showed that the inclusion of other structural genes, especially the full-length ORF3 derived from the virulent strain, could markedly enhance the virulence of attenuated strain resulting in severe diarrhea in infected pigs (13). To clarify this point, further investigation is required to confirm whether the ORF3 could indeed function synergistically with the S protein to determine the pathogenicity of the virus. Furthermore, using a reverse genetics system to generate the infectious-clone-derived PEDV (icPEDV), Beall et al. (25) demonstrated that pigs infected with icPEDVΔORF3 had a substantially lower diarrheic score than those infected with the wild-type PEDV strain PC22A or icPEDV carrying the intact ORF3. It is also notable that the icPEDVΔORF3 is also efficiently transmitted from infected pigs to uninfected pigs via indirect contact leading to disease outcomes (25). These findings altogether suggest the role of PEDV ORF3 as a virulence factor in the *in vivo* model. While the function of ORF3 remains to be determined, mounting evidence points to its strong association with viral virulence. As PEDV targets intestinal epithelial cells for productive infection *in vivo*, this process may require the functional ORF3, possibly via its ion channel activity. However, most *in vitro* studies are based on cultured cells such as Vero, MARC-145, and IPEC-J2, which may not be biologically relevant to what happens *in vivo*. As organoid systems, especially 3D intestinal organoids, could be highly relevant models for assessing differences between PEDV variants, future studies using this platform are needed to gain more insights into the functional role of ORF3.

## The Role of ORF3 in PEDV Replication *in vitro*

Despite its critical role in viral pathogenesis, ORF3 is dispensable for virus growth *in vitro* (25–27). Given that most of the field PEDV strains carry the full-length ORF3 gene in the genome except for only a few isolates harboring a truncation of ORF3 (28, 29), it is likely that the ORF3 may be beneficial for its growth in the natural host. Nonetheless, several studies have reported the inhibitory effects of the ORF3 against the viral growth *in vitro*, especially the recovery of PEDV via reverse genetics (Table 1). It is also important to note that the effect of ORF3 might largely depend on the PEDV variant used in each study. While many studies demonstrated that the intact ORF3 could suppress cell-adapted PEDV replication in cultured cells (27, 31, 32), a few studies showed that the ORF3 could promote viral replication of specific PEDV variants (11, 14) or no effect of the ORF3 at all (25).

**Table 1:** Summary of the effect of ORF3 variants on virus replication and virus virulence.

**PEDV strain**	**ORF3 length (aa)**	**Cell line**	**Virus replication (*in vitro*)**	**Virus virulence (*in vivo*)**	**References**
Attenuated DR13	207	Vero	Higher virus titer than isolated virus	Reduced pathogenicity	(18, 24)
KPEDV-9 P93	207	Vero	N/A	Reduced pathogenicity	(18, 23)
YN P1	224	Vero	PEDV with truncated ORF3 had a higher virus titer than PEDV with FL ORF3.	N/A	(30)
YN P15	144			Virulent	
YN P60	144			N/A	
YN P144	144			Avirulent	
BJ2011C	224	Vero	PEDV with truncated ORF3 had higher growth kinetics than PEDV with FL ORF3.	Highly virulent	(13)
CHM2013	155			Avirulent	
BJ2011C (carrying S from CHM2013)	224			Reduced virulence	
CHM2013 (carrying S from BJ2011C)	155			Reduced virulence	
CHM2013 (carrying the structural-protein from BJ2011C)	224			Increased virulence	
PC22A	224	Vero	PEDV with deleted ORF3 or FL ORF3 had a similar virus titer.	Highly virulent	(25)
ΔORF3 PC22A	Absence			Reduced diarrheic scores	
AVCT_12_	absence	VeroE6-APN^*^	Only PEDV with deleted or truncated ORF3, but not FL ORF3, could be rescued by reverse genetics.	N/A	(27)
AVCT_12_	207 (Δ82-98)				
AVCT_12_	224				
Wild type PEDV CV777	Knockdown ORF3	Vero	Reduced number of viral RNA copies number	N/A	(11)
AH2012/12	223	Vero	Successful virus rescue	N/A	(26)
AVCT_12_ AVCT_12_	224 207 (Δ82-98)	Vero-APN^*^	PEDV with deleted or truncated ORF3 had higher titers than PEDV with FL ORF3.	N/A	(31)
AVCT_12_	Absence				
Field strains Nakorn Phanom 2012 Field strains Nakorn Phanom 2012	207 224	HEK293T	PEDV with truncated ORF3 had higher virus titers than PEDV with FL ORF3	N/A	(32)^**^
Field strains Nakorn Phanom 2014	224				
Field strains Ratchaburi 2014	207				
Field strains Ratchaburi 2014	224				
Attenuated DR13	91 (attenuated DR13)	Vero	PEDV with FL or truncated ORF3 had higher virus titers than PEDV with deleted ORF3.	N/A	(33)
Attenuated DR13	224 (WT DR13)				
Attenuated DR13	224 (WT CV777)				
Attenuated DR13	N/A (field strain NY)				
Attenuated DR13	Absence				
Virulent strain CH/YNKM-8/2013	224	Vero	PEDV with overexpressed ORF3 had higher virus copies (compared within the same strain)	N/A	(14)
Attenuated strain CV777	91				
Attenuated strain AH-M	224				
Virulent strain CH/YNKM-8/2013	224	Vero-ORF3^***^	PEDV with overexpressed ORF3 had higher virus copies (compared within the same strain)	N/A	(14)
Attenuated strain AH-M	91				
Attenuated strain CV777	224				

* *Vero cells stably express porcine aminopeptidase N (pAPN)*.

** *PEDV infectious clone was co-transfected with a plasmid expressing ORF3 variants in HEK293T, and the supernatant was harvested and adsorbed onto VeroE6-APN; the supernatant was collected for virus titration*.

*** *Vero cells stably express PEDV ORF3*.

Thus far, the mechanism by which the ORF3 regulates PEDV replication *in vitro* has been suggested but not yet clearly defined. Wongthida et al. (32) demonstrated that phenylalanine at position 81 (F81) and methionine at position 167 (M167) are possibly responsible for suppressing PEDV growth in the cell culture. A single substitution from phenylalanine to leucine at position 81 (F81L) was sufficient for a productive replication of PEDV carrying the full-length ORF3 in VeroE6-APN cells (32). Likewise, a recent study reported that PEDV strains PC22A, AH2012/12, and CH7 bearing L81 in the ORF3 could be successfully rescued by reverse genetics, underscoring the importance of the amino acid residue 81 in ORF3 in virus adaptation in VeroE6 cells. It is also important to note that the inhibitory effect of ORF3 was also demonstrated in another porcine nidovirus, porcine reproductive and respiratory syndrome virus (PRRSV) (32). This finding leads to the possibility that the PEDV ORF3 might exert its inhibitory activity by affecting the replication step(s) common among nidoviruses.

Despite its inhibitory effect against PEDV reverse genetics rescue, the ORF3 has been shown to enhance PEDV growth in some studies. For example, attenuated PEDV strains, AH-M (bearing truncated ORF3) and CV777 (bearing the full-length ORF3), were reported to grow more efficiently in Vero cells stably expressing ORF3 than those propagated in parental Vero cells (14). Moreover, Si et al. (33) compared the growth kinetics of cell-adapted PEDV (DR13) carrying either intact or truncated ORF3. They found that PEDVs with intact/naturally truncated ORF3 (91 residues) appeared to grow to higher titer than the PEDV lacking the ORF3. While it is still not determined how ORF3 could be regulated to result in different outcomes regarding the PEDV replication, we speculate that this might be partially due to different PEDV strains, variations of PEDV ORF3 length, and cell lines used.

Besides its direct role in PEDV replication, accumulating evidence has suggested that ORF3 might play a role in several cellular processes. It has been shown that ORF3 could regulate the cell cycle progression by prolonging the S phase (14), inhibiting early cell apoptosis (33), and promoting autophagy (34), consequently provide a proper environment for viral propagation. Furthermore, it is also demonstrated that the ORF3 could specifically interact with PEDV S during virus replication (31). This interaction between PEDV proteins suggests that ORF3 might play a pivotal part in the virus life cycle, especially in the natural host (31).

In addition, deletion or truncation of the ORF3 acquired during propagation in cell culture has been used as a genetic marker to differentiate between the field and cell-adapted strains. However, the recombination of a highly pathogenic PEDV and a low pathogenic vaccine strain (30), and a natural PEDV co-infection (35) can result in pathogenic PEDV variants carrying truncated ORF3. These data thus indicate that ORF3 deletion or truncation alone may not be sufficient for safe vaccine candidates. This point underscores the necessity of precise genetic modification on ORF3 combined with other viral genes for vaccine design, which will benefit the surveillance study and disease monitoring in pig farms.

## PEDV ORF3 and Host Interaction

Even though the underlying mechanisms of ORF3 functions related to pathogenicity, virus adaptation, and virus proliferation have not been elucidated, several lines of evidence have emphasized the impact of the interplay between ORF3 and the host's machinery. Microscopic inspection of cells over-expressing the ORF3 showed that the protein was typically localized in the cytoplasm (14, 34, 36), specifically in the perinuclear area and the vesicles (31), the ER, and the Golgi (31, 34, 37). These findings point to the possibility that ORF3 may be associated with distinct cellular pathways that might more or less modulate viral replication and pathogenesis.

A panel of intracellular sorting motif mutations located on the C-terminus of PEDV ORF3 revealed that the YLAI motif (residues 170–173; ^170^YLAI^173^) is essential for the translocation of ORF3 from the ER to the plasma membrane (37). Despite no clear association of ^170^YLAI^173^ with the viral growth and pathogenesis, the finding indicates that the disruption of the ^170^YLAI^173^ signal could affect ORF3 cellular localization and transport, consequently influencing the intracellular interplay with host proteins or the virus itself. By this means, we postulate that the ORF3 with reported C-terminus deletions [91aa (33), 144aa (30), and 155aa (13) length] loss the functional motifs or proper protein folding, leading to disruption of protein trafficking and the interaction with cellular components. Furthermore, an interactome study has demonstrated that ORF3 interacting cellular proteins are enriched in the endo-lysosomal components (endosome, lysosome, and vacuole); many of them are associated with ubiquitination and immune signaling pathways (JAK/STAT and interleukin signaling) (36). In particular, the binding of ORF3 and vacuolar protein-sorting-associated protein 36 (VPS36), a component of the endosomal sorting complexes required for transport II (ESCRT-II), was found to trigger ORF3 degradation, which, in turn, suppressed PEDV replication in VeroE6 cells (36). In addition, ORF3 was shown to modulate the NF-κB signaling pathway to downregulate proinflammatory cytokines (interleukin-8; IL-8 and TNF-α). Interestingly, ORF3 could also interact with the IκB kinase β (IKBKB), resulting in the up-regulation of the IKBKB-meditated NF-κB promoter activity but down-regulation of the IKBKB-meditated IFN-β promoter and IFN-β mRNA expression (38). Consistently, Wu et al. (39) also demonstrated that ORF3 could inhibit the NF-κB activation by hampering nuclear factor p65 phosphorylation and nuclear translocation as well as down-regulating p65 expression, resulting in a reduction of proinflammatory cytokines IL-6 and IL-8 production. Therefore, these findings highlight the role of ORF3 in governing host immune responses that account for virus growth and disease severity.

PEDV has been reported to induce apoptosis by activating mitochondrial apoptosis-inducing factors (40) and accelerating autophagy associated with inflammatory cytokine expression (41). Notably, ORF3 was also shown to localize in ER and trigger ER stress by increasing the expression level of GRP78 and activating the PERK-eIF2α signaling pathway and inducing autophagy *in vitro* (34). These findings point to a possible link between PEDV infection/ORF3 and mitochondria. Even though the contribution of ORF3 in PEDV-induced apoptosis has not been well-defined, Si et al. (33) demonstrated that ORF3 and its naturally truncated form could inhibit apoptosis through the suppression of procaspase-3 activation to facilitate virus replication. However, conflicting results of ORF3-induced apoptosis have been reported in studies using ORF3-expressing cells and cells infected with the PEDV bearing ORF3 (33, 34).

**Figure 2:**
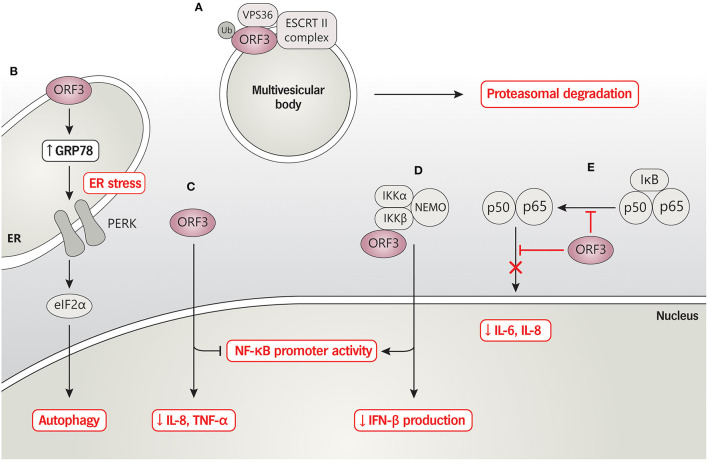
PEDV ORF3 and host protein interaction. **(A)** ORF3 protein interacts with VPS36 leading to ORF3 degradation via proteasomal degradation pathway. **(B)** PEDV ORF3 induces the expression level of GRP78 and activates the PERK-eIF2α signaling pathway resulting in enhancing cell autophagy. **(C)** ORF3 inhibits NF-κB promoter activity and down-regulates IL-8 and TNF-α mRNA expression while **(D)** induces NF-κB promoter activity and reduces IFN-β production in the binding with an overexpressed-IKBKB. **(E)** ORF3 suppresses phosphorylation and nuclear translocation of NF-κB, resulting in reduced proinflammatory cytokines IL-6 and IL-8.

The current data thus suggest that, besides being an ion channel or viroporin, ORF3 could interact with a large number of host's proteins as a means to manipulate cellular machinery to regulate virus replication and pathogenesis. Thus, characterization of specific amino acids or functional domains would be beneficial to precisely modify the PEDV ORF3 gene as a principle for vaccine design concerning virus cultivation and attenuation. A summary of the interaction of ORF3 and cellular proteins, based on current published data, is depicted in Figure 2.

## Prospects of ORF3 for PEDV Vaccine Design

Serial passages in host cells to obtain PEDV vaccine candidates often result in the loss or truncation of the ORF3 gene. For example, Chen et al. (30) showed that when the virulent PEDV strain YN was subjected to multiple passages in Vero cells, a high-growth PEDV with shortened ORF3 (144 residues) emerged during viral propagation as early as passage 15, designated YN15. However, the YN15 was still highly pathogenic in piglets, suggesting that shortening of ORF3 is not sufficient for virus attenuation (30). Interestingly, when YN15 was further propagated in Vero cells up to 144 passages, the virus, termed YN144, showed a markedly attenuated phenotype with no further mutations in the ORF3 gene (30). Likely, modification of ORF3 and mutations of critical residues especially of the spike protein, are crucial for developing a PEDV-based live virus vaccine. Comparing the effect of PEDV ORF3 variants (full-length vs. various truncated forms) in distributing viral pathogenesis in the identical viral genetic background would give rise to solid evidence of its role.

A reverse genetic system is a powerful tool for molecular studies and vaccine development. Given that ORF3 has been shown to have inhibitory effects on virus rescue, understanding its mechanism and region(s) of amino acid sequence responsible for this particular aspect can help overcome difficulties in PEDV isolation *in vitro*. As mentioned earlier, the presence of ORF3 in different forms can markedly influence PEDV virus rescue by reverse genetics and virus pathogenicity in the natural host. Extensive deletion of ORF3 might likely support efficient virus rescue and propagation in Vero cells. However, a high-growth virus with deleted ORF3 could poorly infect intestinal cells *in vivo*, affecting the virus's property to be used as an oral vaccine. Therefore, the strategy of ORF3 gene engineering for virus vaccine production should be considered to enable the virus to grow well in cell culture and, at the same time, to become less virulent but still maintaining its infectivity in intestinal cells.

## Conclusions and Future Studies

ORF3 is a multi-functional protein that plays essential roles in modulating cellular mechanisms, particularly the host immune system and apoptosis. These roles of ORF3 are likely associated with virus replication and pathogenicity. Due to the lack of comprehensive studies of ORF3 and inconsistent findings of ORF3 among available literature, its impacts on virus adaptation in cell culture, virus replication, and pathogenesis are still not well-elucidated. Future studies utilizing various approaches are needed to gain more precise insights into our understanding of this protein. For example, a series of PEDV ORF3 mutations (variants) should be constructed together with the S protein to generate recombinant PEDVs to show the effect of each mutation in the virus phenotype. The impact of ORF3 variants on PEDV growth kinetics should be investigated in detail *in vitro* and *in vivo*. The mechanistic role of ORF3 in interferon signaling pathway/inflammasome activation could be further explored. Knowledge obtained from these studies would be a groundwork for viral gene mutagenesis to improve a better live-attenuated PEDV vaccine accomplishing high yield in cell culture with a minimum passaging, induction of specific immune response, and no reversion to virulence in the vaccinated animal.

## Author Contributions

All authors listed have made a substantial, direct and intellectual contribution to the work, and approved it for publication.

## Funding

This work was supported by National Vaccine Institute (NVI), Thailand, under grant number P2150344.

## Conflict of Interest

The authors declare that the research was conducted in the absence of any commercial or financial relationships that could be construed as a potential conflict of interest.

## Publisher's Note

All claims expressed in this article are solely those of the authors and do not necessarily represent those of their affiliated organizations, or those of the publisher, the editors and the reviewers. Any product that may be evaluated in this article, or claim that may be made by its manufacturer, is not guaranteed or endorsed by the publisher.
